# Myc-Foxo, a link between glutaminolysis and autophagy

**DOI:** 10.1186/1753-6561-6-S3-P6

**Published:** 2012-06-01

**Authors:** Sheri Zola, Paola Bellosta

**Affiliations:** 1 Dept of Genetics and Developments, Columbia University Med Center, New York, NY, 10032, USA

## 

Our recent observations that dMyc expression in the *Drosophila* FB -a metabolic tissue with similar physiological functions as mammalian adipose tissue and liver – remotely influences the development of the whole animal, allows us to explore its function in a living growing organism. dMyc in FB increases animal survival, which correlates with an increased level of autophagy, especially visible in FB from animals under starvation. Our preliminary data show that in addition to enzymes responsible for glycolysis, dMyc upregulates in FB the expression of the putative glutamine transporter slcA7/minidiscs and of glutaminase, the enzyme that converts glutamine to alfaketoglutarate with the production of ammonia. By varying the nutrient availability, we found that dMyc expression induces the accumulation of high levels of ammonia in the supernatant of S2 cells. Ex-vivo cultures of FB incubated in these supernatants exhibited autophagy, suggesting that dMyc is responsible for the expression of soluble proautophagic factors, possibly ammonia. In FB autophagy is particularly evident during starvation, conditions where dFOXO transcription sustains dMyc expression. Using genetic manipulation of components of glutamine metabolism we are currently exploring if the relationship between dMyc and dFOXO in FB affects ammonia production and autophagy via glutamine catabolism. With these experiments we aim to understand whether FOXO regulates Myc to induce glutamine signaling, placing FOXO-function relevant for glutaminolysis, which is one of the most relevant survival pathways in tumor cells.

